# Educational Inequalities in Obesity among Mexican Women: Time-Trends from 1988 to 2012

**DOI:** 10.1371/journal.pone.0090195

**Published:** 2014-03-05

**Authors:** Carolina Perez Ferrer, Anne McMunn, Juan A. Rivera Dommarco, Eric J. Brunner

**Affiliations:** 1 Research Department of Epidemiology and Public Health, University College London, London, United Kingdom; 2 Centro de investigación en Nutrición y Salud, Instituto Nacional de Salud Pública, Cuernavaca, México; Universidad Peruana Cayetano Heredia, Peru

## Abstract

**Background:**

Obesity is one of the leading causes of global morbidity and mortality. Trends in educational inequalities in obesity prevalence among Mexican women have not been analysed systematically to date.

**Methods:**

Data came from four nationally representative surveys (1988, 1999, 2006, and 2012) of a total of 51 220 non-pregnant women aged 20 to 49. Weight and height were measured during home visits. Education level (higher education, high school, secondary, primary or less) was self-reported. We analysed trends in relative and absolute educational inequalities in obesity prevalence separately for urban and rural areas.

**Results:**

Nationally, age-standardised obesity prevalence increased from 9.3% to 33.7% over 25 years to 2012. Obesity prevalence was inversely associated with education level in urban areas at all survey waves. In rural areas, obesity prevalence increased markedly but there was no gradient with education level at any survey. The relative index of inequality in urban areas declined over the period (2.87 (95%CI: 1.94, 4.25) in 1988, 1.55 (95%CI: 1.33, 1.80) in 2012, trend p<0.001). Obesity increased 5.92 fold (95%CI: 4.03, 8.70) among urban women with higher education in the period 1988–2012 compared to 3.23 fold (95%CI: 2.88, 3.63) for urban women with primary or no education. The slope index of inequality increased in urban areas from 1988 to 2012. Over 0.5 M cases would be avoided if the obesity prevalence of women with primary or less education was the same as for women with higher education.

**Conclusions:**

The expected inverse association between education and obesity was observed in urban areas of Mexico. The declining trend in relative educational inequalities in obesity was due to a greater increase in obesity prevalence among higher educated women. In rural areas there was no social gradient in the association between education level and obesity across the four surveys.

## Introduction

Obesity is one of the leading causes of global mortality and morbidity[1]. It is associated with cardiovascular disease, diabetes, some cancers such as oesophagus, pancreatic, colorectal and postmenopausal breast cancer, and mortality [2]–[5]. Obesity prevalence has increased dramatically in all regions of the world including the poorest nations [6]. Inequalilties in obesity will translate into inequalities in morbidity and mortality.

In developed countries, there tends to be an inverse association between obesity prevalence and socioeconomic position (SEP), especially among women, while in developing countries the association is direct [7], [8]. Generally, as a country develops economically, obesity increases faster among the more disadvantaged women compared to the more advantaged ones and inequalities emerge and widen [9]–[14]. In China and Brazil, for example, inequalities in obesity became larger in the 1980s and 1990s because BMI increased faster among the least educated Chinese women and the poorest Brazilian women compared to more advantaged women [13], [14]. A different trend has been observed in the United States and Canada where the initial inverse association between SEP and obesity attenuated over the 1980s and 1990s especially among women [15], [16]. This was due to greater increases in the prevalence of obesity in more advantaged compared with less advantaged groups [15]–[18].

Mexico is an upper middle income country that experienced a rapid nutrition transition, from a traditional to a Western pattern food supply. Obesity prevalence among adult women trebled in 25 years [19]. Mexico ranks second behind the United States in a 2010 OECD report ranking 40 countries according obesity prevalence [20]. The overall increase in obesity prevalence in Mexico has been similar to that experienced by industrialized countries like the United States[21]–[23] and greater than that experienced by other middle income countries like Brazil [24].

It is not well understood how obesity is distributed across socioeconomic groups and to what extent inequalities in obesity are changing in Mexico. Previous studies assessing the association between socioeconomic position and obesity in Mexico have used one wave of cross-sectional data and have found an inverse association in urban areas and a direct or non-linear association in rural areas [25]–[29]. The aim of this study was to evaluate the trend in inequalities in obesity by education level for adult Mexican women for the period 1988–2012, utilizing four waves of nationally representative data. We assess whether Mexico follows the characteristic middle income country trend, where inequalities in obesity emerge and increase as the country transitions, or if it follows the North American trend, where relative inequalities have declined. In addition, we explore heterogeneity in the nutrition transition within Mexico by analysing findings for urban and rural areas separately.

## Methods

### Data sources

Data were extracted from four nationally representative cross-sectional surveys conducted in 1988, 1999, 2006 and 2012[30]–[33]. All were designed to collect information on nutrition and the latter two on health and health related services and interventions (National Health and Nutrition Survey). The first two surveys focused on women ages 12 to 49 and children. The last two focus on men and women age 20 and older, children and adolescents. We selected women age 20 to 49 years old as our study population because this group was measured in the four surveys. The design of the sample was similar in all surveys and included stratification and probabilistic selection of clusters in different stages. Stratification variables included degree of urbanicity (except for 1988) and socioeconomic factors. The primary sampling units (PSU; municipal subdivisions) were defined across the entire country. A sample of PSUs was selected in each stratum at state level, with probability proportional to population size. Secondary sampling units (SSU), smaller geographic units within each PSU, were defined and a sample of these was selected following the same procedures. Within SSUs a given number of households were selected. Within each household all women were interviewed and measured in the 1988 and 1999 survey or one woman was randomly selected to be interviewed and measured in the 2006 and 2012 surveys. Each individual in the dataset carries a weight which represents the inverse probability of being sampled adjusted for survey non-response.

Response rates at household level ranged from 80% to 97%. The achieved sample of households was in the range 13 263 in 1988 to 50 528 in 2012. The total number of women with demographic information across the four surveys was n = 60 331. Missing values for BMI in the achieved sample of women were n = 1 035 (8.6%), n = 2 857 (18.2%), n = 3 575 (20.3%) and n = 560 (3.7%) respectively for each survey. The 1999 and 2006 datasets did not distinguish between women who refused to be measured and those not selected to be measured. Missingness due to refusal to be measured is lower than the overall missingness level in these surveys. Missing values for education and other covariates were all <5%. Cases with missing values were excluded after careful examination of missing data patterns suggested that selection bias in the main findings was minimal. Missing BMI was not associated with perception of being overweight or obese, and perception of being overweight or obese was highly correlated with measured overweight or obesity (Spearman ρ = 0.55, p<0.001) in the survey with the largest proportion of missing data (2006). After exclusion of missing data and extreme, implausible values for BMI (BMI<10, BMI>75; less than 0.5% of total sample) our analytical sample consisted of *n* = 51 220 non-pregnant, 20 to 49 year old women.

### Ethics statement

Written consent was obtained from adults participating in the study, including the parents or tutor of children. Verbal consent was obtained from children. The study protocol, data collection instruments and consent forms and procedures were approved by the ethics committee of the National Institute of Public Health in Mexico.The present study was based on an anonymous, public-use data set with no identifiable information on the study participants.

### Outcome, exposure and covariates

Body mass index (BMI) was calculated as weight (kg) divided by the square of height (m^2^). Obesity was defined as a BMI≥30 kg/m^2^. Height and weight were measured using standard procedures by trained health teams during home visits [19], [30]–[32]. The main exposure variable was level of education defined as self-reported attendance to higher education, high school, secondary, or primary education or less. These categories refer to well-known milestones in the Mexican education system. Age (in years) was included as an adjustment covariate in all models, given the linear association of age with BMI. Area of residence has been identified as effect modifier of the association between education and obesity in previous studies [34], thus analyses are stratified by this variable. Urban areas were defined as communities with more than 2 500 inhabitants and rural areas with less than 2 500 inhabitants.

### Statistical Analysis

A pooled dataset of the four surveys was created. All analyses were adjusted for survey design and weighted using the STATA 12 survey commands (svy). We first computed the age-standardised obesity prevalence in each survey. We then calculated the age standardised prevalence of obesity by education group in each survey. The Mexican 2000 census population was used as the standard population. The linear trend in the education gradient was assessed in a regression where the outcome was obesity, the exposure education as a continuous variable, adjusted for age [35], [36]. Deviation from linearity in the education gradients was tested by adding a quadratic term to the model. We used generalised linear models (log binomial regression) rather than logistic regression as has been recommended when modelling frequent outcomes [35], [36]. Generalised linear models estimate the prevalence ratio.

The relative index of inequality is a standard summary measure of inequality. It is recommended when making comparisons of health inequality over time or across populations [37].The slope index of inequality measures absolute inequalities using similar methodology. To estimate these measures of inequality, education level was transformed onto a scale from 0 (highest level of education) to 1 (lowest level of education) and weighted to reflect the share of the population at each educational level by calculating the midpoint of the proportion in the population in each category. This was done separately for urban and rural areas of each survey wave. For example, 16.2% of the study participants in urban areas in 2006 were in the higher education group and 20.8% were in the high school group. Participants in the higher education group were assigned a score of 0.08 (0.16/2) meaning 92% of the urban population of 2006 had lower education than the average person in this group. Those in the high school group were assigned a score of 0.27 (0.16+(0.21/2)) and so on for each education level [38]. To obtain the relative index of inequality and slope index of inequality, obesity was regressed on the new education variable in a model adjusted for age. We used generalised linear models, with a logarithmic link function to calculate the relative index of inequality (rate ratio) and with an identity link function to calculate slope index of inequality (rate difference)[39]. The relative index of inequality is interpreted as the prevalence *ratio* between the two ends of the educational hierarchy – obesity prevalence at the bottom divided by obesity prevalence at the top. The slope index of inequality is the prevalence *difference* between top and bottom. Using the slope index of inequality and the total population (weighted expanded sample), excess obese cases in the most disadvantaged groups were estimated for urban areas. Linear trends of the relative index of inequality and slope index of inequality over the period were tested by estimating the p value for an interaction term between education and years since baseline, i.e. 1988 survey was coded 1, 1999 11 and so on, to account for the different time intervals between surveys. The model was adjusted for age in addition to year and education rank [40], [41].

Relative increases in obesity prevalence over time by education level were estimated by generalised linear models where obesity was the dependent variable and survey year was the independent variable [13]. Analyses were stratified by education level, and age was used as a covariate. These models estimate an age-adjusted prevalence ratio that reflects the magnitude and statistical significance of the increase in obesity prevalence by education level in the period 1988–2012. Mantel-Haenszel x^2^ tests for homogeneity were calculated to assess statistical differences in obesity prevalence ratios across education levels in urban and rural areas[42]. Absolute increases in obesity prevalence by education level were estimated by linear regression using the same stratification and adjustment variables described above. These models estimate an age adjusted prevalence difference by education level in the period 1988–2012.

### Sensitivity analysis

In order to test whether inequalities in obesity differ between and within birth cohorts over time, we compared the relative index of inequality trends for an older, less educated and a younger, more educated cohort over the period 1999–2012 among women living in urban areas. A variable for year of birth was created by subtracting the age of the woman from the year of the survey. Individuals born between 1963 and 1979 were included because they had data available for three time points (1999, 2006 and 2012; n = 17 695). Two “pseudo cohorts” were created, the older cohort (women born between 1963 and 1971, n = 9 031) and the younger cohort (women born between 1972 and 1979, n = 8 664). The education rank variable was constructed again with the education proportions in the two cohorts. The relative index of inequality was calculated as previously described for each period stratified by cohort. The trend of the relative index of inequality for each cohort was then estimated and compared using a three way interaction term composed of education rank, year and cohort [14]. This analysis examined whether the inequality trend in urban areas differed by cohort.

Further sensitivity analyses were conducted, fitting models to estimate relative index of inequality and slope index of inequality adjusted for height, given that height was inversely correlated with BMI and directly correlated with education, and using obesity class II and III (BMI≥35) as the outcome.

## Results

The proportion of urban population in the survey samples was 83.7%, 75.5%, 77.0% and 78.6% for 1988, 1999, 2006 and 2012 respectively. Except for 1988, the proportion of urban dwellers in each sample was similar to the nearest census estimate[43]. Table 1 presents selected characteristics of the study population according to survey year. The average age of women increased from 32 to 33 from 1988 to 2012, there was no age difference between urban and rural areas. In urban areas the proportion of women who entered higher education doubled to 23% between 1988 and 2012 and those with primary education or less halved to 23%. In rural areas there were smaller improvements in participation. The average weight of Mexican women increased by 12 kg in urban areas and 10 kg in rural areas in the period 1988–2012 while height increased by 1 cm in urban areas and remained constant in rural areas. Height was socially patterned in both urban and rural areas (Table S1). The change in height from 1988 to 2012 was not statistically significant for any education level for urban or rural areas. Obesity prevalence more than trebled in the 24 year period with urban areas being especially affected. The largest increase took place in the period 1988–1999. A third of the population in this demographic group was obese in 2012.

**Table 1:** Age standardised distribution of women for selected characteristics 1988–2012.

	URBAN				RURAL			
	1988	1999	2006	2012	1988	1999	2006	2012
**Complete cases, N**	8 887	8 205	9 906	9 588	1 315	4 308	4 068	4 943
**Age, mean**	32.4 (0.1)	33.8 (0.1)	34.0 (0.1)	33.8 (0.1)	32.2 (0.3)	33.8 (0.1)	33.7 (0.2)	33.4 (0.2)
**Age group, %**								
20–24.9	22.8 (0.6)	17.6 (0.5)	17.3 (0.6)	18.3 (0.7)	22.6 (1.5)	18.5 (0.6)	17.4 (0.9)	18.5 (0.9)
25–29.9	19.2 (0.5)	16.5 (0.5)	16.5 (0.6)	16.2 (0.6	20.1 (1.2)	15.9 (0.6)	17.1 (0.7)	19.0 (0.9)
30–34.9	17.6 (0.6)	17.8 (0.5)	17.7 (0.6)	19.5 (0.6)	17.3 (1.2)	17.2 (0.7)	19.0 (0.8)	18.6 (0.7)
35–39.9	16.5 (0.5)	19.5 (0.6)	18.3 (0.6)	16.9 (0.6)	17.1 (0.8)	19.3 (0.7)	17.6 (0.8)	16.3 (0.7)
40–44.9	13.0 (0.5)	16.8 (0.6)	16.5 (0.6)	15.0 (0.5)	12.3 (1.4)	16.9 (0.7)	17.2 (0.9)	14.4 (0.6)
45–49.9	10.8 (0.4)	11.8 (0.5)	13.6 (0.6)	14.1 (0.6)	10.6 (1.0)	12.3 (0.6)	11.7 (0.8)	13.3 (0.6)
**Level of education, %**								
Higher education	10.2 (0.6)	14.6 (0.6)	16.2 (0.8)	22.6 (0.8)	2.2 (0.6)	1.8 (0.3)	2.4 (0.4)	6.5 (0.7)
High school	17.4 (0.7)	20.5 (0.6)	20.8 (0.7)	22.7 (0.7)	8.4 (1.6)	5.8 (0.6)	5.6 (0.7)	13.0 (0.8)
Secondary	17.0 (0.6)	24.3 (0.6)	28.8 (0.8)	31.2 (0.8)	9.4 (1.4)	13.7 (0.8)	24.6 (1.2)	33.9 (1.3)
Primary or less	55.4 (1.3)	40.6 (0.8)	34.4 (0.9)	23.4 (0.8)	79.9 (3.2)	78.8 (1.2)	67.5 (1.3)	46.6 (1.4)
**Weight (kg), mean**	56.3 (0.2)	64.4 (0.2)	66.5 (0.2)	68.1 (0.3)	54.6 (0.7)	59.7 (0.4)	63.5 (0.4)	64.8 (0.3)
**Height (cm), mean**	153.5 (0.2)	153.6 (0.1)	154.2 (0.1)	154.9 (0.1)	152.0 (0.4)	150.5 (0.2)	151.9 (0.2)	152.4 (0.2)
**BMI, mean**	23.9 (0.1)	27.3 (0.1)	28.0 (0.1)	28.4 (0.1)	23.6 (0.2)	26.3 (0.1)	27.5 (0.1)	27.8 (0.1)
**Obese, %**	9.5 (0.39)	26.3 (0.6)	30.9 (0.68)	34.5 (0.76)	8.1 (1.22)	21.3 (0.83)	27.9 (1.12)	30.7(0.99)

Standard errors in parenthesis.

**Figure 1:**
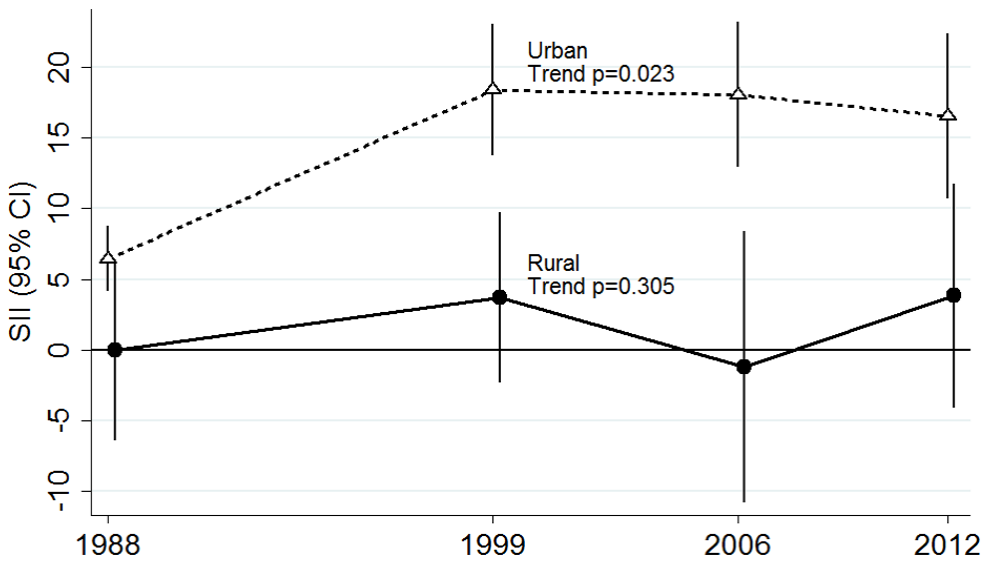
Trend in absolute inequalities in obesity for urban and rural Mexican women 1988–2012. Each point represents the slope index of inequality (SII) for the particular year. Error bars represent the 95% confidence intervals of the SII. Plotted estimates are adjusted for age.

**Figure 2:**
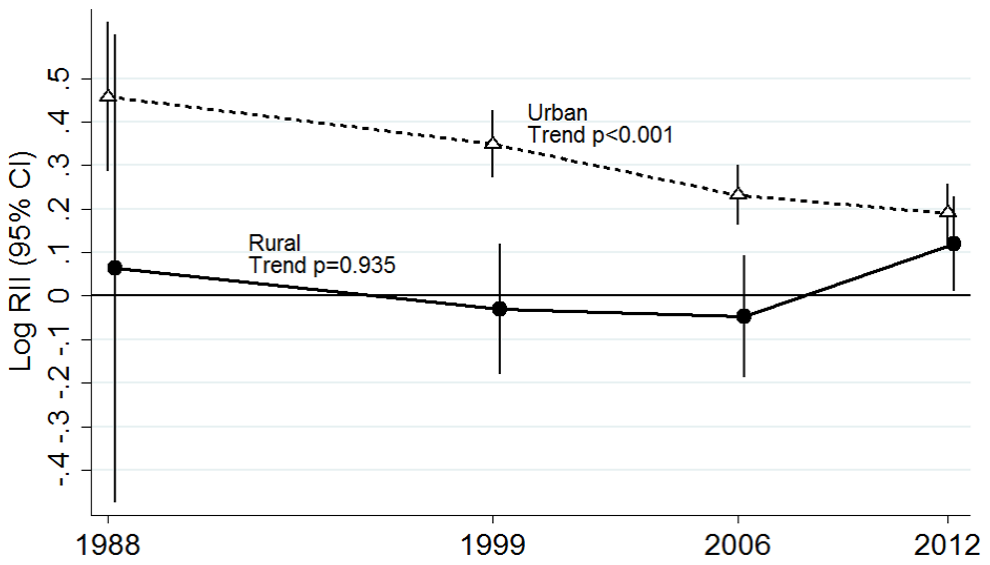
Trend in relative inequalities in obesity for urban and rural Mexican women 1988–2012. Each point represents the relative index of inequality (RII) for the particular year. Error bars represent the 95% confidence intervals of the RII. Plotted estimates are adjusted for age.

There was an inverse linear association between obesity and education in urban areas (Table 2). One step lower education level was associated with 32% higher obesity prevalence (prevalence ratio (PR) 1.32 95%CI 1.19, 1.46) in 1988 and by 12% (PR 1.12 95%CI 1.08, 1.17) in 2012. Absolute inequalities measured by the slope index of inequality increased from 6.4 (95%CI 4.1, 8.8) in 1988 to 18.36 (95% CI 13.70, 23.03) in 1999 and then levelled off (Table 3 and Figure 1). Excess obesity cases in women with primary education or less ranged from over 300,000 in 1988 to over a million in 1999 (table 3). Relative inequalities decreased. The relative index of inequality in urban areas was 2.87 (95% CI: 1.94, 4.25) in 1988 and declined over the period to 1.55 (95% CI: 1.33, 1.80) in 2012, trend p<0.001 (Table 3 and Figure 2). After adjusting for height, the trend coefficient changed by less than 1% and statistical significance was unaltered. In rural areas, obesity prevalence increased markedly overall but there was no gradient with education level at any survey wave, and both the relative index of inequality and slope index of inequality were non-significant. However, there is evidence of a significant non-linear variation in obesity prevalence by education level in some survey waves (Table 2). The same declining trend in relative inequalities in urban areas and no trend in rural areas is observed when the outcome is class II and III obesity (BMI≥35) (Table S2).

**Table 2:** Age standardised obesity prevalence by education level stratified by urban and rural areas.

	1988		1999		2006		2012	
	%	95% CI	%	95% CI	%	95% CI	%	95% CI
**Urban areas**								
Higher education	5.05	(3.09,7.00)	17.77	(14.9,20.66)	21.79	(18.43,25.15)	26.70	(23.76,29.65)
High school	7.03	(5.22,8.83)	21.32	(18.80,23.83)	26.77	(23.91,29.63)	33.65	(30.30,37.0)
Secondary	8.43	(6.63,10.24)	24.78	(22.50,27.09)	32.32	(29.72,34.91)	36.59	(34.13,39.06)
Primary or no education	11.18	(10.09,12.27)	31.65	(29.60,33.70)	36.45	(34.13,38.78)	38.52	(35.24,41.80)
**Rural areas**								
Higher education	3.67	(0.96,6.38)	14.51	(7.12,21.89)	27.83	(19.89,35.78)	21.57	(15.27,27.88)
High school	5.38	(0.27,10.48)	20.17	(16.64,25.69)	24.91	(16.40,33.41)	28.79	(23.94,33.65)
Secondary	14.34	(5.64,23.04)	30.44	(26.50,34.37)	31.52	(26.81,36.22)	32.26	(28.81,35.71)
Primary or no education	8.10	(5.56,10.63)	21.62	(19.76,23.50)	27.64	(24.63,30.66)	31.02	(28.00,34.04)

**Table 3:** Absolute and relative inequalities in obesity.

	Urban		Rural	
	RII (95%CI)	SII (95%CI)	RII (95%CI)	SII (95%CI)
1988	2.87*(1.94,4.25)	6.44*(4.12,8.77)	1.16 (0.34,3.98)	−0.04 (−6.0,6.0)
1999	2.22*(1.86,2.66)	18.36*(13.70,23.03)	0.93 (0.66,1.32)	4.0 (−2.3,9.7)
2006	1.71*(1.45,2.00)	18.03*(12.91,23.15)	0.90 (0.65,1.24)	−1.2 (−11,8.0)
2012	1.55*(1.33,1.80)	16.52*(10.72,22.33)	1.13 (0.89,1.44)	4.0 (−4.0,11.0)
Linear trend across surveys p	p<0.001	p = 0.023^a^^,^ b	p = 0.935	p = 0.305^a^

RII: Relative index of inequality.

SII: Slope index of inequality.

*p<0.001 in each survey year.

a estimated using survey weighted linear regression.

b quadratic term p<0.001.

Absolute increases in obesity prevalence between 1988–1999 were greater among women with primary or less education in both urban and rural areas, compared with women with higher education. From 1999 to 2012 absolute increases in obesity prevalence were similar for all education groups therefore the slope index of inequality remains largely unchanged as ilustrated in figure 1. In contrast, relative increases where largest in the most educated women in urban areas (p<0.001 for the null hypothesis of homogeneity of rates across education levels). Obesity increased 5.92 fold (95% CI 4.03, 8.70) among urban women with higher education in the period 1988–2012 compared to 3.23 fold (95%CI 2.88, 3.63) for urban women with primary or no education (Table 4). Between 2006 and 2012, the prevalence of obesity among urban women with secondary education, primary or less did not increase significantly, while there was a 22% increase in obesity prevalence among women with high school or higher education (PR 1.22 p<0.05 for both groups). This resulted in the stepwise decline from 1988 to 2012 in the relative index of inequality as illustrated in figure 2. Among rural women, there appears to be larger increases in the prevalence of obesity over time in the group with high school education (PR 6.96 95%CI 2.92, 16.55) however homogeneity in the rates across education levels could not be rejected.

**Table 4:** Absolute and relative increases in obesity prevalence by education level from 1988 to 2012.

	Relative increase		Absolute increase	
	1988–2012		1988–2012	
	PR^a^	95% CI	%^b^	95% CI
***Urban areas***				
Higher education	5.92*	(4.03, 8.70)	20.41	(17.04, 23.78)
High school	5.45	(4.14, 7.16)	25.39	(21.72, 29.06)
Secondary	4.74	(3.81, 5.91)	27.37	(24.47, 30.27)
Primary or no educ	3.23	(2.88, 3.63)	29.29	(26.17, 32.40)
***Rural areas***				
Higher education	4.82†	(0.90, 25.80)	16.49	(7.19, 25.78)
High school	6.96	(2.92, 16.55)	20.58	(14.93, 26.22)
Secondary	3.16	(1.61, 6.22)	17.57	(11.56, 23.57)
Primary or no educ	3.70	(2.64, 5.18)	24.30	(20.43, 28.17)

a Age adjusted prevalence ratio.

b Age adjusted prevalence difference.

Test for homogeneity across education levels *p<0.001 † p = 0.50.

### Sensitivity analysis

There was a large shift in the distribution of attained level of education between the two birth cohorts. The younger cohort was significantly more educated than the older cohort; among the younger cohort 28.2% (95% CI 26.7, 29.6) had primary education or less vs. 39.8% (95%CI 38.2,41.4) among the older cohort (F = 38.4 p<0.001).

Table 5 shows the relative index of inequality trend from 1999 to 2012 in urban areas stratified by birth cohort. For the older cohort, there was a non-significant tendency towards declining inequality similar to that described in the unstratified analysis. There was no trend in the relative index of inequality for the younger cohort. The older and younger cohort relative index of inequality trends were significantly different from each other (p = 0.005).

**Table 5:** Relative index of inequality (RII) stratified by birth cohort in urban areas.

	Survey year			
Birth cohort	1999	2006	2012	trend p
	RII (95% CI)	RII (95% CI)	RII (95% CI)	
Older 1963–1971	2.31* (1.77,3.00)	1.71* (1.35, 2.17)	1.61* (1.25,2.06)	0.062
Younger 1972–1979	1.63∼ (1.02,2.61)	2.06* (1.46,2.92)	1.39* (1.10,1.74)	0.179

*p<0.001.

∼ p<0.05.

## Discussion

Our study is the first to examine time trends in inequalities in obesity among Mexican women using a unified analytic method. Previous studies used single waves of data, showing an inverse association between education and obesity in urban areas and a direct or non-linear association in rural areas [25]–[29]. Obesity prevalence among Mexican women increased dramatically across all education groups over the period 1988–2012 with the largest increases between 1988 and 1999. Although the difference in obesity prevalence between urban and rural areas was not large, the social patterning of obesity differed significantly. There was an inverse association between education level and obesity prevalence among urban-dwelling but not rural-dwelling Mexican women. In urban areas there was strong evidence that relative inequalities in obesity declined over the period 1988–2012 as a consequence of a larger increase in obesity prevalence in more educated compared to less educated women.

In urban areas, where most Mexican women live, obesity has disproportionately affected those with the least education for the last 25 years. The declining trend in relative educational inequalities observed is similar to that in North America, and differs from the female obesity inequality trend typical of other low and middle income countries [10], [15], [17]. We tested whether this trend could be the result of differential changes in height across education groups over the period but found no evidence for this. Absolute inequalities increased from 1988 to 2012. In 2012, based on PAR and assuming the excess obesity prevalence was preventable, 744 437 obesity cases could have been avoided if the lowest education group had the same health experience as those in the highest education group.

In rural areas there was no educational gradient at any survey wave. Women with primary education or less had a lower prevalence of obesity than more educated women at the first two survey waves. More recently, obesity prevalence in women with primary education or less has caught up with the prevalence of more advantaged groups. It is likely that women with primary education or less were protected from obesity by their limited resources in the earlier period of the surveys. As living conditions improved and low-cost processed food and calorific drink products penetrated rural areas [44], disadvantaged women lost this protection. Speculatively, there may be a crossover to an inverse association between education and obesity in the near future, as has been observed in numerous middle income countries [25]. In Mexico, economic development has concentrated in urban areas [45] and it is likely that the nutrition transition is lagging behind in rural areas [46].

This study uses education as an indicator of socioeconomic position. The meaning of education may differ for the older and younger birth cohorts studied here, with differing distributions of knowledge, skills and opportunities that affect health [47]. We tested whether the period variation in the effect of education on obesity reflected a change in the meaning of education for the different cohorts. This was not supported by our findings. The birth cohort stratified analysis suggested that the protective role of education varied within the same cohort over the years. The cohort stratified analysis also showed that although the younger cohort was significantly more educated, it was not more protected from obesity than the older cohort. These observations led us to believe that changes to environmental or cultural factors cutting across all socioeconomic groups had a more powerful effect on women's choices and possibilities, reducing the protective effect of personal characteristics such as education [15].

The food and built environment in Mexico changed substantially over the 1980s and 1990s leading to changes in diet, increase in calorie intake and a decrease in physical activity and energy expenditure[48]. Highly processed foods became widely available partly due to a 25 fold increase in foreign direct investment to the Mexican food industry from 1989 to 1999 [49] facilitated by the signing of the North American free trade agreement (NAFTA) in 1994. Over this period, women's participation in the labour force also increased substantially from 17% in the 70s to 43% in 2010 [50_ENREF_52]. This may have caused considerable changes in food purchase and preparation patterns at household level contributing to increased calorie intake [51_ENREF_53]. Physical activity at a population level has likely decreased as a result of urbanisation, changes in occupation, car ownership, increased time spent watching television and using computers. These changes affected the entire population and might explain the large increases in obesity. Speculatively, these environmental changes may have affected SEP groups differentially.

Although the literature suggests that in general those most affected by increased availability of processed foods are disadvantaged SEP groups [52_ENREF_54,53_ENREF_55], this does not seem to be the case in Mexico. Our study suggests that increases in obesity prevalence were greatest for women with more education especially in urban areas. Women with more education might have been the first to access processed/North American foods in the early days of market liberalization. These foods were novel and added variety to the traditional Mexican diet. Chains selling energy dense foods and beverages that target upper middle income groups have had high growth. For example convenience stores targeting urbanites with limited time, have grown at a rate close to 1000 new stores per year during the 1990s and 2000s [49]. Working women, who have tended to be more educated than those who do not work [50_ENREF_52], may have relied more heavily on convenience foods with the consequence of increasing calorie intakes.

The trends in inequalities in obesity prevalence in Mexico may be an exception to the middle income country pattern and particular to Mexico, USA and Canada due to their unique relationship. NAFTA has facilitated market integration with North America and promoted a regional food system [54_ENREF_56]. Demographically there are extensive social networks between the USA and Mexico due to immigration. Mexican migrants in the USA send remittances and also social and cultural norms back to Mexico [55_ENREF_57]. Similar environmental factors may be shaping the social distribution of obesity in the three countries.

### Strengths and Limitations

Our study strengths include using nationally representative data from comparable health surveys. Height and weight were measured by trained personnel and the main exposure, attendance to education, is minimally prone to recall bias. Education is frequently used as an indicator of SEP in low and middle income countries; its use allows comparability with previous studies. This study also has limitations. We performed a complete cases analysis, losing observations in each survey. Missing data patterns were examined carefully. Women with missing BMI were more educated, richer and younger than those with complete data. It is likely that missingness is due to operational issues such as health teams not visiting some households or women not being available to be measured due to work or study. It is less likely the refusal was associated with their weight, based on an analysis of perceived weight. For the two middle surveys, missing values reported are likely to be an overestimation of true missingness given that the datasets do not distinguish between women who refused or could not be measured and those not selected to be measured. The proportion of urban/rural dwellers from the 1988 sample is significantly different to the 1980 census estimate (66.3% urban; 33.7% rural). The 1988 survey was the first nutrition survey to ever be undertaken in Mexico and did not stratify by urban and rural dwelling in the sampling design. The representativeness of findings especially for rural areas in this survey is thus a limitation. Our analysis was limited to education level as indicator of socioeconomic position. A woman's education is strongly determined by her parental socioeconomic position [47,56_ENREF_58] and because it is set at an early age, it is not sensitive to changes in SEP thereafter. The pattern of inequalities in obesity could be different if other socioeconomic position measures such as income or wealth are used because each indicator is associated with obesity through different pathways. It was beyond the scope of this study to study other exposures. It was not possible to carry out a more robust cohort effect analysis because the data do not span enough years for each birth cohort. As more surveys are carried out in Mexico in the future, cohort effects in inequality trends could be further explored. Lastly, the cross sectional nature of the data precludes exploration of causal directions in the relationship between SEP and obesity.

### Conclusions

Obesity increased substantially in Mexico across all education groups in both urban and rural areas over the study period. In urban areas, the most disadvantaged women have the largest burden of obesity however, relative educational inequalities decreased from 1988 to 2012. This was due to higher increases in obesity among women with high school or higher education compared with women with primary education or less. In rural areas there was no educational gradient in obesity prevalence. These findings have important implications for public health nutrition policy in Mexico and suggest that structural and population-wide approaches to obesity prevention may be as important as targeting high risk groups.

## Supporting Information

Table S1 Mean height by education level 1988–2012.
(DOCX)

Table S2 Age standardised class II and III obesity (BMI≥35) prevalence by education level and summary inequality measures 1988–2012.
(DOCX)
