# Correction: Educational Inequalities in Obesity among Mexican Women: Time-Trends from 1988 to 2012

**DOI:** 10.1371/journal.pone.0097394

**Published:** 2014-05-05

**Authors:** The PLOS ONE Staff

Several in-text references are incorrect in both the PDF and XML versions of the article. These errors were introduced during the preparation of the article for publication. Please see below for a list of the incorrect references and their corrections.

In the 5^th^ paragraph of the Discussion, [50_ENREF_52] should be [50] and [51_ENREF_53] should be [51].

In the 6^th^ paragraph of the Discussion, [52_ENREF_54,53_ENREF_55] should be [52,53] and [50_ENREF_52] should be [50].

In the 7^th^ paragraph of the Discussion, [54_ENREF_56] should be [54] and [55_ENREF_57] should be [55].

In the Discussion sub-section titled “Strengths and Limitations,” [47,56_ENREF_58] should be [47,56]
