# Supplementary material for: Educational Inequalities in Obesity among Mexican Women: Time-Trends from 1988 to 2012
Source: PLoS One. 2014 Mar 5;9(3):e90195. doi: 10.1371/journal.pone.0090195 (PMC3943903; doi:10.1371/journal.pone.0090195)
Supplement: Table S1 — Mean height by education level 1988–2012. (DOCX) [file pone.0090195.s001.docx]

**Supporting information for: Educational inequalities in obesity among Mexican women: time-trends from 1988 to 2012**

**Table S1: Mean height by education level**

|  | **1988** | **1999** | **2006** | **2012** |
| --- | --- | --- | --- | --- |
| **Urban areas** |  |  |  |  |
| Higher education | 157.4 (0.3)* | 156.5 (0.3) | 157.0 (0.3) | 157.3 (0.2) |
| High school | 156.0 (0.2) | 155.2 (0.2) | 155.6 (0.2) | 155.6 (0.2) |
| Secondary school | 154.4 (0.2) | 153.8 (0.2) | 154.1 (0.2) | 154.7 (0.2) |
| Primary or less | 151.7 (0.2) | 151.6 (0.1) | 152.2 (0.2) | 152.3 (0.3) |
| *Linear trend (p)* | *-1.98 (<0.001)* | *-1.65 (<0.001)* | *-1.66 (<0.001)* | *-1.68 (<0.001)* |
|  |  |  |  |  |
| **Rural areas** |  |  |  |  |
| Higher education | 156.0 (1.0) | 155.3 (0.5) | 154.4 (0.9) | 156.6 (0.6) |
| High school | 154.5 (0.7) | 153.8 (0.5) | 155.6 (0.5) | 154.0 (0.4) |
| Secondary school | 154.1 (0.7) | 152.5 (0.5) | 153.2 (0.3) | 153.4 (0.3) |
| Primary or less | 151.5 (0.5) | 149.9 (0.2) | 150.9 (0.3) | 150.6 (0.3) |
| *Linear trend (p)* | *-1.49 (<0.001)* | *-2.11 (<0.001)* | *-2.17 (<0.001)* | *-2.08 (<0.001)* |

*Height in cm, standard error in parenthesis
