# Supplementary material for: Educational Inequalities in Obesity among Mexican Women: Time-Trends from 1988 to 2012
Source: PLoS One. 2014 Mar 5;9(3):e90195. doi: 10.1371/journal.pone.0090195 (PMC3943903; doi:10.1371/journal.pone.0090195)
Supplement: Table S2 — Age standardised class II and III obesity (BMI≥35) prevalence by education level and summary inequality measures 1988–2012. (DOCX) [file pone.0090195.s002.docx]

**Supporting information for: Educational inequalities in obesity among Mexican women: time-trends from 1988 to 2012**

**Table S2: Age standardised class II and III obesity (BMI ≥35) prevalence by education level and summary measures of inequality**

|  | **1988** | | **1999** | | **2006** | | **2012** | |
| --- | --- | --- | --- | --- | --- | --- | --- | --- |
|  | **%** | **95% CI** | **%** | **95% CI** | **%** | **95% CI** | **%** | **95% CI** |
| **Urban areas** |  |  |  |  |  |  |  |  |
| **Sample N** | **8 887** |  | **8 205** |  | **9 906** |  | **9 588** |  |
| Higher education | 1.17 | (0.09, 2.25) | 5.29 | (3.70, 6.88) | 8.05 | (5.80, 10.30) | 9.59 | (7.46, 11.71) |
| High school | 1.36 | (0.73, 1.99) | 5.55 | (4.38, 6.73) | 8.60 | (6.83, 10.37) | 10.70 | (8.34, 13.05) |
| Secondary | 1.73 | (1.03, 2.43) | 7.97 | (6.44, 9.50) | 11.88 | (10.21, 13.55) | 13.77 | (12.00, 15.54) |
| Primary or no education | 2.76 | (2.20, 3.33) | 10.74 | (9.35, 12.12) | 13.58 | (12.07, 15.09) | 14.10 | (11.80, 16.40) |
| ***Summary measures of inequality*** |  |  |  |  |  |  |  |  |
| *Slope index of inequality* | *2.00** | *(0.94, 3.08)* | *7.17** | *(4.64, 9.69)* | *6.55** | *(3.45, 9.65)* | *7.03** | *(3.00, 11.10)* |
| *SII linear trend across surveys*^a^ | *p=0.008* |  |  |  |  |  |  |  |
| *Relative index of inequality* | *4.31** | *(2.05, 9.04)* | *2.82** | *(2.01, 3.94)* | *1.89** | *(1.40, 2.55)* | *1.84** | *(1.36, 2.48)* |
| *RII linear trend across surveys* | *p=0.002* |  |  |  |  |  |  |  |
|  |  |  |  |  |  |  |  |  |
| **Rural areas** |  |  |  |  |  |  |  |  |
| **Sample N** | **1 315** |  | **4 308** |  | **4 068** |  | **4 943** |  |
| Higher education | 3.67 | (0.96, 6.38) | 2.86 | (-1.33, 7.05) | 11.72 | (4.60, 18.83) | 6.38 | (2.88, 9.88) |
| High school | 0.62 | (-0.45, 1.69) | 8.34 | (3.42, 13.25) | 15.47 | (7.56, 23.39) | 9.58 | (6.36, 12.79) |
| Secondary | 2.30 | (-0.65, 5.25) | 9.08 | (6.01, 12.15) | 7.86 | (5.86, 9.86) | 11.83 | (9.59, 14.07) |
| Primary or no education | 1.94 | (0.95, 2.93) | 5.48 | (4.59, 6.37) | 7.51 | (6.22, 8.80) | 8.37 | (6.88, 9.85) |
| ***Summary measures of inequality*** |  |  |  |  |  |  |  |  |
| *Slope index of inequality* | -1.31 | (-4.42, 1.80) | -8.06 | (-4.30, 2.68) | -5.40* | (-10.00, -0.88) | -2.30 | (-6.95, 2.27) |
| *SII linear trend across surveys^a^* | p=0.171 |  |  |  |  |  |  |  |
| *Relative index of inequality* | 0.60 | (0.12, 3.03) | 0.66 | (0.32, 1.36) | 0.56* | (0.33, 0.94) | 0.78 | (0.51, 1.17) |
| *RII linear trend* | p=0.898 |  |  |  |  |  |  |  |

*p<0.001 in each survey year

^a^ estimated using survey weighted linear regression
